# Two Cases of Human Papillomavirus-Related Oropharyngeal Cancer After the Treatment of Cervical Cancer

**DOI:** 10.7759/cureus.8434

**Published:** 2020-06-04

**Authors:** Tomoko Yamazaki

**Affiliations:** 1 Head and Neck Cancer: Medical Oncology, Miyagi Cancer Center, Natori, JPN

**Keywords:** hpv-related oropharyngeal cancer, cervical cancer, double cancer, human papillomavirus

## Abstract

Recently, the incidence of human papillomavirus (HPV)-related head and neck cancers (oropharynx and oral) has increased rapidly. Secondary HPV-related cancers after an initial index cancer at a site associated with an HPV infection have been reported, including invasive cervical, vaginal, vulval, anal, penile, and oropharyngeal cancer.

Here, we describe two female oropharyngeal cancer patients who had been treated for secondary cervical cancers with chemoradiotherapy and achieved a good clinical response.

## Introduction

The incidence of human papillomavirus (HPV)-related oropharyngeal cancer (OPC) has recently increased worldwide [1]. Patients with HPV-related OPC tend to be younger and have lower rates of tobacco and alcohol use than patients with HPV-unrelated OPC. The prognosis for HPV-related OPC is better than that for HPV-unrelated disease [1-2]. The latest Union for International Cancer Control (UICC)/American Joint Committee on Cancer (AJCC) ver. 8 tumor, node, metastasis (TNM) staging of OPC thus distinguished between HPV-related and HPV-unrelated OPC [3-4].

According to the World Health Organization (WHO), HPV is now the most common sexually transmitted infection worldwide, with approximately 5% of all cancers being caused by HPV [5-6]. Although patients diagnosed with primary HPV-related cancers are often cured, they remain at risk for secondary HPV-related malignancies, necessitating the careful follow-up of these patients [7].

## Case presentation

Case 1

**Figure 1:**
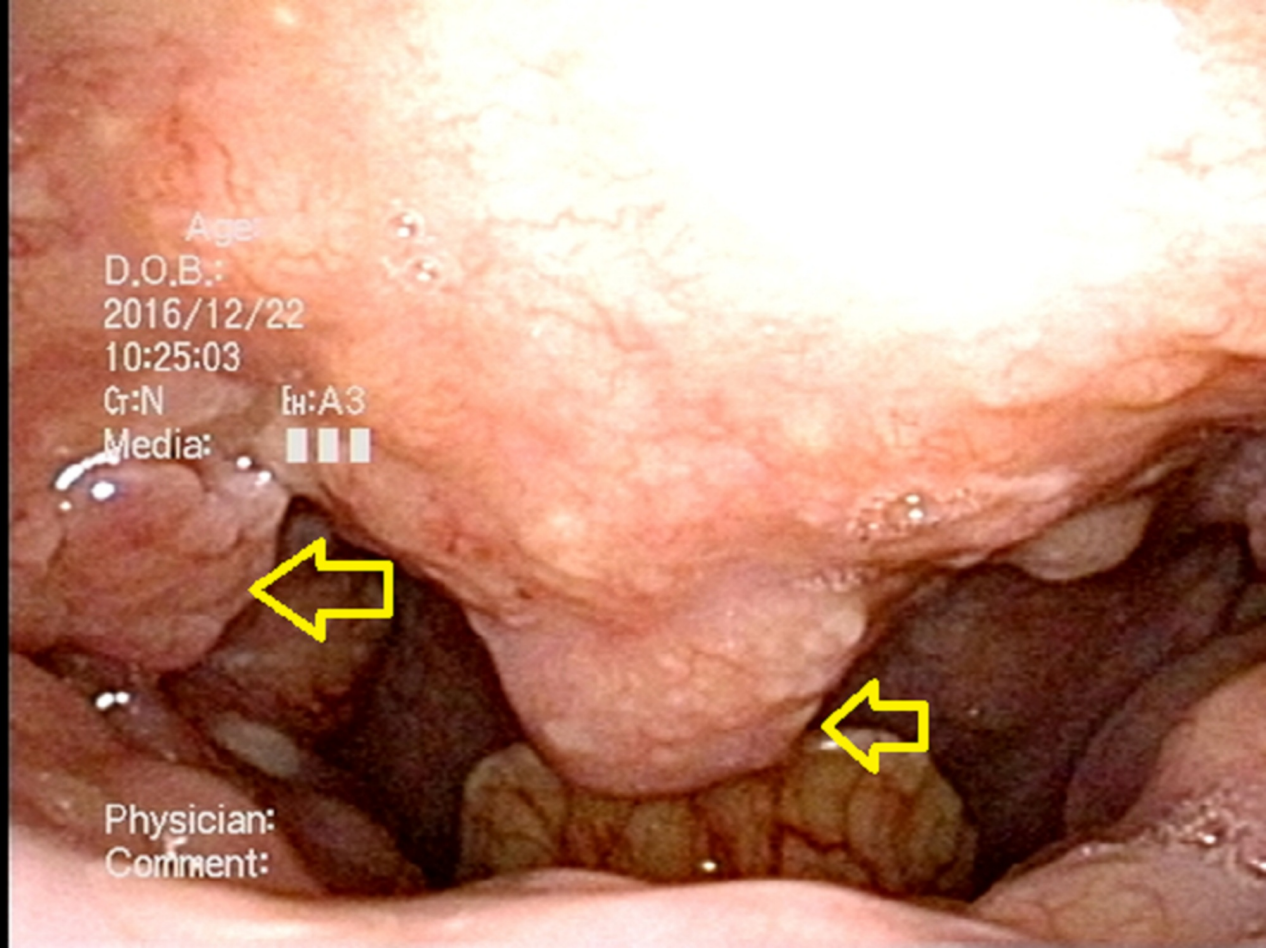
Case 1; The primary site of fiberscopic laryngoscopy A tumor extending from the tonsil to the soft palate on the right side

**Figure 2:**
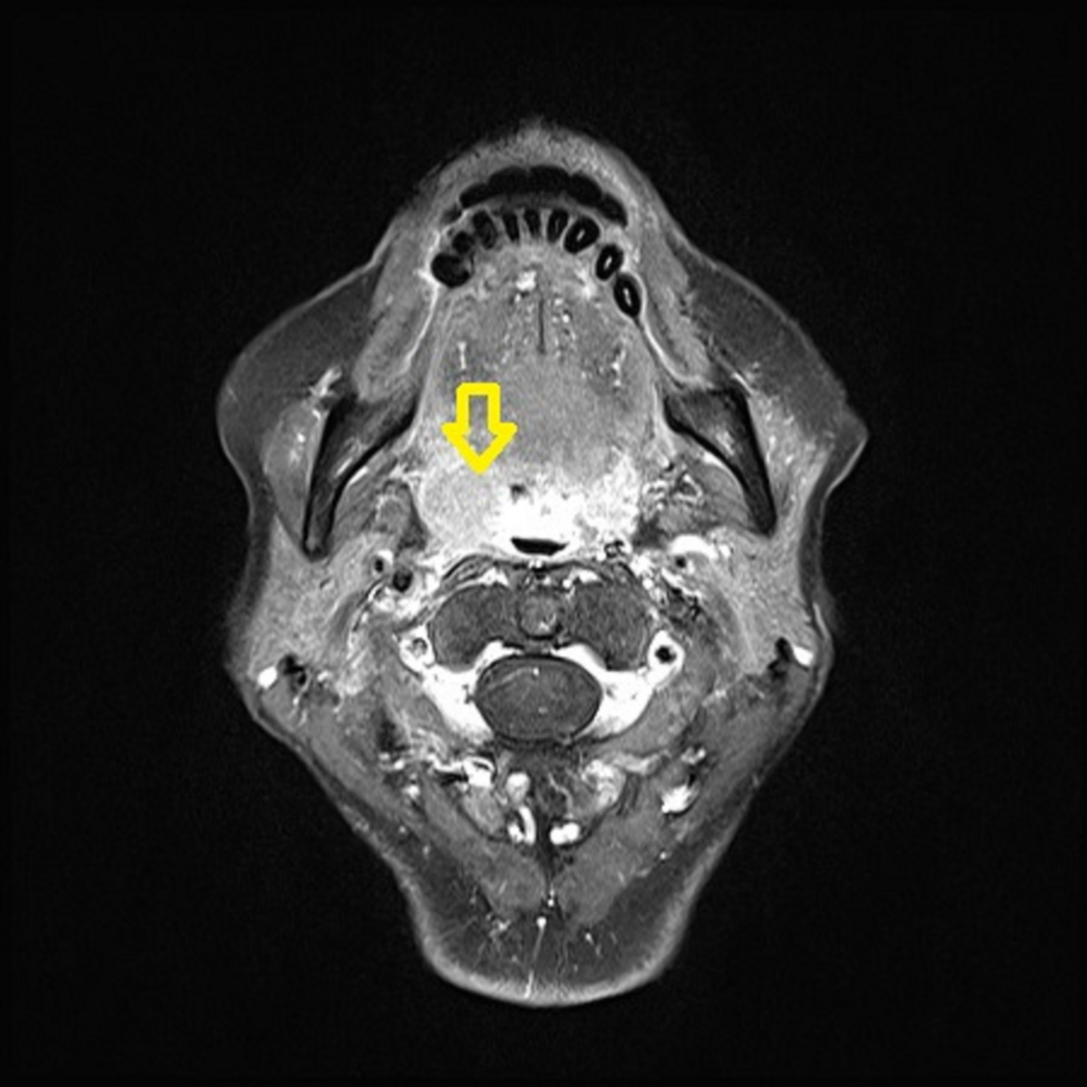
Case 1; head and neck MRI before CRT The tumor is located to the right in the oropharynx; size is about 3 cm MRI: magnetic resonance imaging; CRT: chemoradiotherapy

A 69-year-old female with HPV-related OPC was referred to our hospital in December 2016. She had suffered from HPV type 16-related cervical cancer at age 58 years and was treated with surgery followed by adjuvant chemoradiotherapy. She had had no recurrence. She was neither a smoker nor a drinker. She felt painless cervical lymph nodes on the right side in August 2016 and visited a local otolaryngologist in December 2016. Fiberoptic laryngoscopy revealed a tumor extending from the tonsil to the soft palate on the right (Figure 1). Biopsy of the oropharyngeal lesion confirmed well-differentiated squamous cell carcinoma of the right soft palate. A magnetic resonance imaging (MRI) scan confirmed a primary lesion of approximately 3 cm extending from the right tonsil to the soft palate on the right with bilateral enlarged cervical and retropharyngeal lymph nodes, from levels II to IV and Vb. Their maximum diameter was approximately 2 cm. There was no evidence of extranodal infiltration (Figure 2). Computed tomography (CT) of the chest, abdomen, and pelvis revealed no abnormalities.

She was staged as clinical T2N2cM0 (stage IVA, AJCC 7th edition 2010, Stage II, AJCC 8th edition 2018, HPV IHC[+], p16/INK4a gene expression [++], type 16 positive and type 18 positive). At the tumor board meeting, it was decided that she should receive cisplatin (CDDP)-based chemoradiotherapy (CRT). Following the placement of percutaneous endoscopic gastrostomy (PEG), CRT (intensity-modulated radiation therapy (IMRT) 70 Gy/35 fractions and CDDP 80 mg/m^2^ for two cycles) were initiated in February 2017. As she developed Grade 3 neutropenia and leukopenia, chemotherapy was delayed, but she was able to complete radiation therapy without radiation interruption. Radiation dermatitis was Grade 2 and mucositis was Grade 3. After the treatment was completed, she required tube feeding via PEG. Following rehabilitation, she could eat orally after two months. She achieved a complete response and has remained alive since then.

Case 2

**Figure 3:**
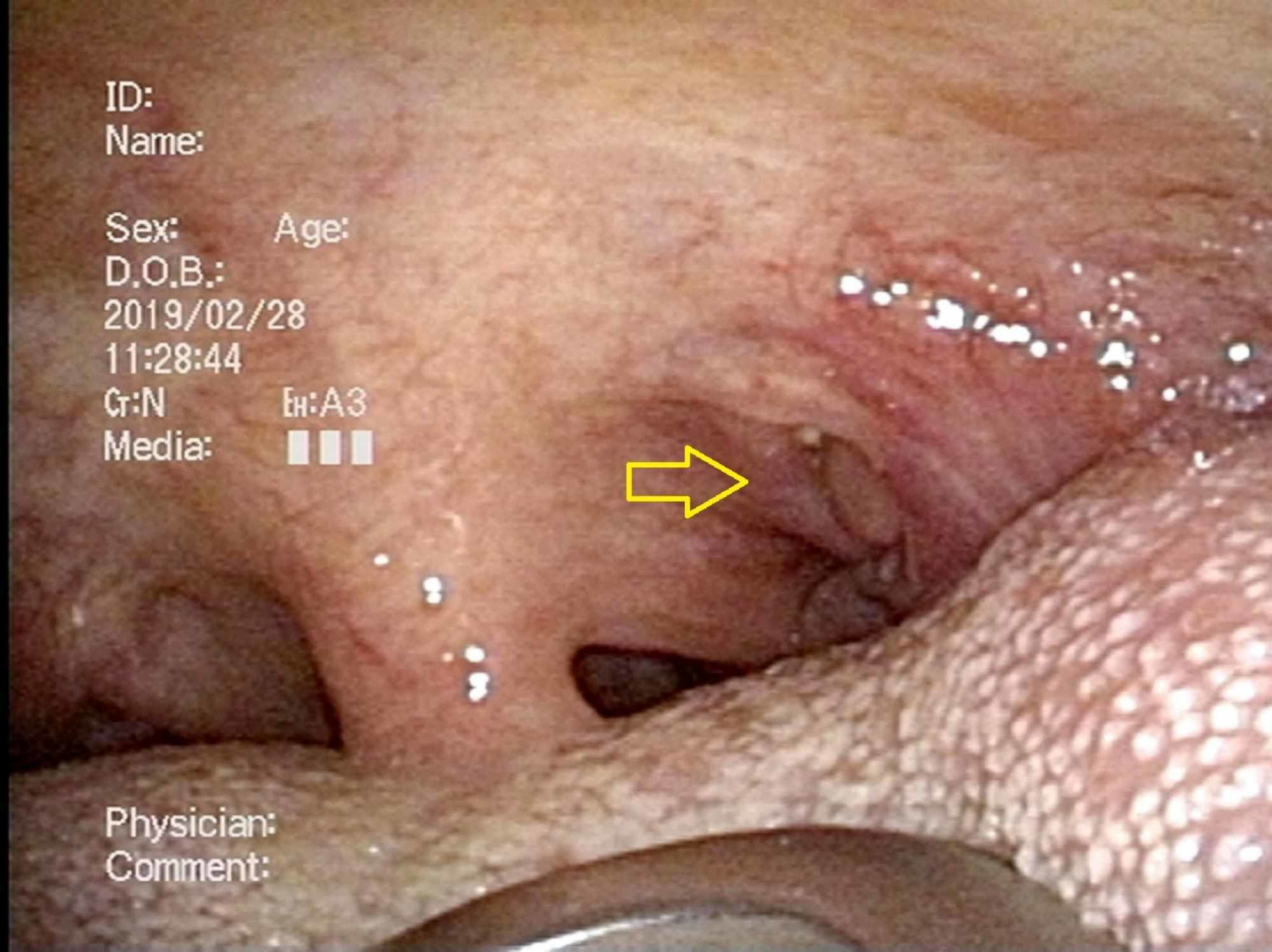
Case 2; the primary site of the fiberscopic laryngoscopy Reddish discoloration on the left tonsil

A 70-year-old female with HPV-related OPC was referred to our hospital in December 2018. She had suffered from cervical cancer at 36 years of age and was treated successfully with surgery. The HPV type was unknown because the operation was performed 34 years ago. She was neither a smoker nor a drinker. She noted painless neck nodes on the left and visited a local otolaryngologist in December 2018. Fiberoptic laryngoscopy revealed reddish discoloration on the left tonsil (Figure 3). Biopsy of the lesion confirmed well-differentiated squamous cell carcinoma of the left tonsil.

**Figure 4:**
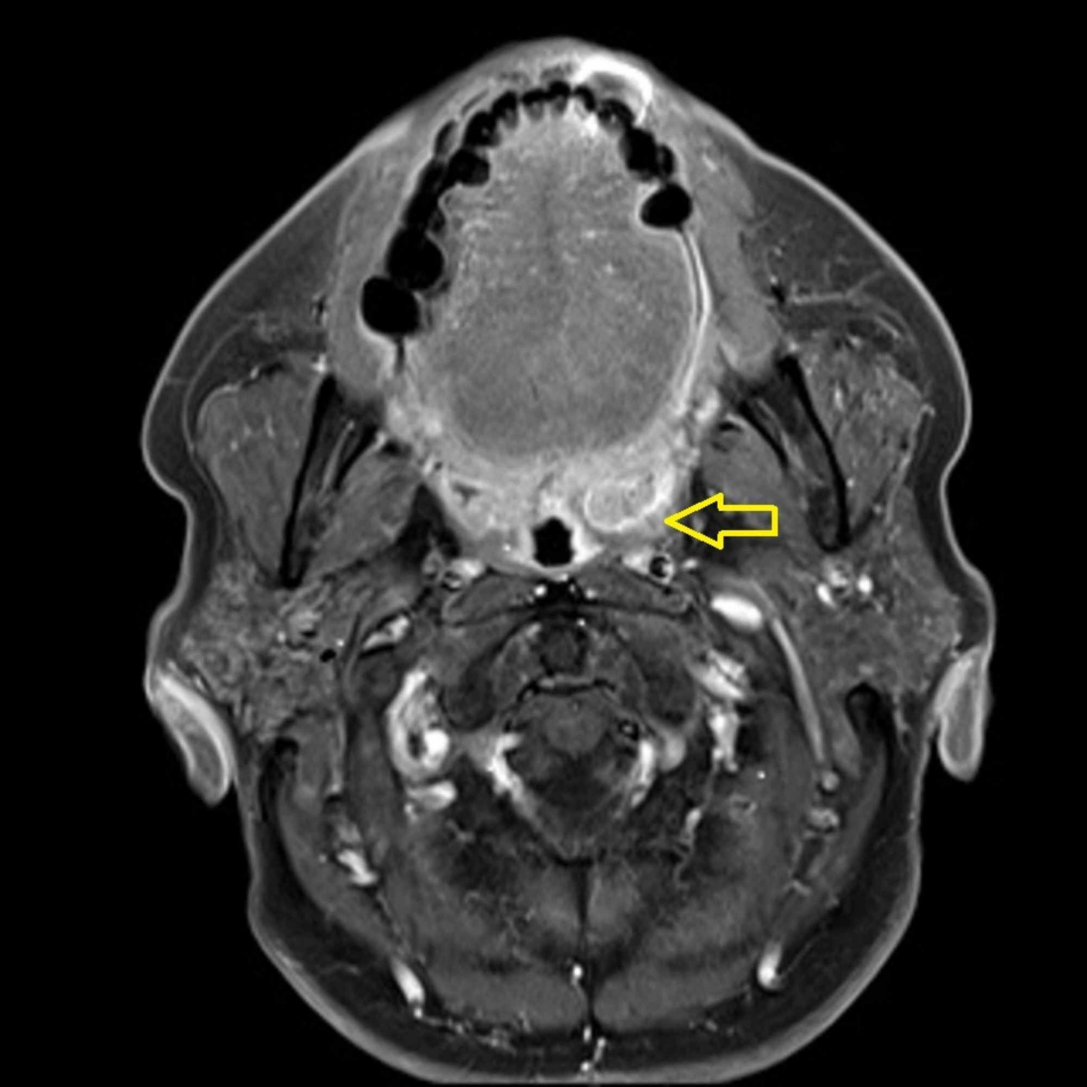
Case 2; head and neck MRI before CRT The tumor is located on the left tonsil; size is about 3 cm MRI: magnetic resonance imaging; CRT: chemoradiotherapy

Findings on the magnetic resonance imaging (MRI) scan were of the primary tonsillar lesion measuring approximately 3 cm. The lymphadenopathy was defined as level III on the left side. The maximum lymph node diameter was approximately 3 cm; there was no extranodal infiltration (Figure 4). She was staged as clinical T2N1M0 (Stage I, AJCC 8th edition 2018), HPV IHC [+], p16/INK4a gene expression [++], type 16 positive, and type 18 negative.

She received PEG and underwent CRT in January 2019. IMRT was 70 Gy/35 fractions and CDDP 80 mg/m^2^ for three cycles. The treatment was completed with only one adverse event, Grade 3 mucositis, requiring tube feeding via PEG. Following rehabilitation, she could eat orally after two months. She achieved a complete response, has had no recurrence, and remains alive at nine months after treatment.

## Discussion

Here, we present two patients with remote histories of successfully treated cervical cancer who presented with new-onset HPV-positive OPC.

Approximately 5% of all cancers worldwide are caused by HPV, including 90%-93% of anal cancers, 12%-63% of OPCs, 36%-40% of penile cancers, 40%-60% of vaginal cancers, and 40%-51% of vulvar cancers [7-8]. The incidence of HPV-related OPC has risen rapidly in recent years [9-10]. According to repository data from the Surveillance, Epidemiology, and End Results (SEER) program, the prevalence of HPV-negative cancers decreased by 50% from 1988 to 2004, while that of HPV-positive OPC increased by 225%[11]. HPV-related OPC patients have a better prognosis than those with HPV-unrelated disease. HPV-related OPC patients also tend to be younger and healthier, with a higher income and no history of tobacco or alcohol abuse [12-13]. In comparison, patients with HPV-unrelated OPC tend to be heavy smokers and drinkers.

However, if healthy, young, nonsmoking patients with symptoms such as painlessly enlarged neck lymph nodes fail to seek medical attention, the appropriate diagnosis and treatment will be delayed. Psychological distress is also reportedly more frequent among patients with HPV-related than those with HPV-unrelated OPC, while head and neck cancers are associated with the highest levels of distress among oncology patients, with high incidences of major depression and anxiety [5,14-15]. Head and neck cancer survivors are almost twice as likely to die from suicide than survivors of other cancers, indicating the need to include mental health professionals in multidisciplinary teams involved in the care of patients with head and neck cancer. In addition, all patients with head and neck cancer require surveillance for possible recurrence and secondary primary cancers. Given that patients with HPV-related OPC tend to be younger, they require particular support and meticulous care.

The seventh edition of the AJCC/UICC TNM staging system did not consistently distinguish between prognostic subgroups for HPV-related and non-HPV-related OPC. However, the eighth edition, which does subclassify according to HPV status, came into effect on or after January 1, 2017 [3-4].

HPV immunostaining is now required in patients with OPC. Although it does not detect HPV directly, p16 is considered a surrogate marker, and p16 immunohistochemistry is economical and convenient, with high sensitivity [16].

HPV-associated-cervical cancer is classified into high-risk and low-risk groups, among which HPV types 16, 18, 31, 33, 35, 39, 45, 51, 52, 56, 58, 59, 68, 73, and 82 are considered high risk [17]. HPV infection is very common and women who acquire the virus sexually remain infected throughout their lives. About 70% of newly acquired HPV infections disappear within one year and about 90% within two years [18]. However, HPV infection for >12 continuous months increases the risk of cervical cancer.

A meta-analysis found that patients with any HPV-related first primary cancer were at an increased risk of developing a second primary cancer such as cervical cancer. A second primary cancer may involve the same or a different area as the primary cancer [19].

We considered if HPV-related OPC was caused by the same HPV types as cervical cancer. Case 1 received diagnoses of both types of cancer. However, although Case 2 had OPC caused by HPV 16, she had been treated for cervical cancer 34 years ago at another hospital and the samples were unavailable.

There is currently no routine examination system for head and neck cancer in Japan. Most patients visit a local doctor when they have symptoms such as neck lymph node swelling, pain, and difficulty swallowing and are only referred to institutions such as ours when their conditions have already worsened. Recent studies suggested that vaccination may protect against oral HPV infection, and vaccinated adults had a lower prevalence of HPV infection in both the oral cavity and other areas as compared with unvaccinated adults [20]. We, therefore, suggest that all children should receive HPV vaccination in Japan.

## Conclusions

In conclusion, HPV-related cancer (for example, cervical, anal, vaginal, and so on) patients should be examined regularly, for a long time. In addition, we have to impart correct information about HPV and HPV vaccination.
